# Expenditures and Health Care Utilization Among Adults With Newly Diagnosed Low Back and Lower Extremity Pain

**DOI:** 10.1001/jamanetworkopen.2019.3676

**Published:** 2019-05-10

**Authors:** Lily H. Kim, Daniel Vail, Tej D. Azad, Jason P. Bentley, Yi Zhang, Allen L. Ho, Paras Fatemi, Austin Feng, Kunal Varshneya, Manisha Desai, Anand Veeravagu, John K. Ratliff

**Affiliations:** 1 Department of Neurosurgery, Stanford University School of Medicine, Stanford, California; 2 Quantitative Sciences Unit, Stanford University School of Medicine, Stanford, California

## Abstract

**Importance:**

Low back pain (LBP) with or without lower extremity pain (LEP) is one of the most common reasons for seeking medical care. Previous studies investigating costs in this population targeted patients receiving surgery. Little is known about health care utilization among patients who do not undergo surgery.

**Objectives:**

To assess use of health care resources for LBP and LEP management and analyze associated costs.

**Design, Setting, and Participants:**

This cohort study used a retrospective analysis of a commercial database containing inpatient and outpatient data for more than 75 million individuals. Participants were US adults who were newly diagnosed with LBP or LEP between 2008 and 2015, did not have a red-flag diagnosis, and were opiate naive prior to diagnosis. Dates of analysis were October 6, 2018, to March 7, 2019.

**Exposures:**

Newly diagnosed LBP or LEP.

**Main Outcomes and Measures:**

The primary outcome was total cost of care within the first 6 and 12 months following diagnosis, stratified by whether patients received spinal surgery. An assessment was performed to determine whether patients who did not undergo surgery received care in accordance with proposed guidelines for conservative LBP and LEP management. Costs resulting from use of different health care services were estimated.

**Results:**

A total of 2 498 013 adult patients with a new LBP or LEP diagnosis (median [interquartile range] age, 47 [36-58] years; 1 373 076 [55.0%] female) were identified. More than half (55.7%) received no intervention. Only 1.2% of patients received surgery, but they accounted for 29.3% of total 12-month costs ($784 million). Total costs of care among the 98.8% of patients who did not receive surgery were $1.8 billion. Patients who did not undergo surgery frequently received care that was inconsistent with clinical guidelines for LBP and LEP: 32.3% of these patients received imaging within 30 days of diagnosis and 35.3% received imaging without a trial of physical therapy.

**Conclusions and Relevance:**

The findings suggest that surgery is rare among patients with newly diagnosed LBP and LEP but remains a significant driver of spending. Early imaging in patients who do not undergo surgery was also a major driver of increased health care expenditures. Avoidable costs among patients with typically self-limited conditions result in considerable economic burden to the US health care system.

## Key Points

**Question:**

What are the nationwide patterns of health care utilization in patients newly diagnosed with low back and lower extremity pain?

**Findings:**

In this cohort study of nearly 2.5 million US patients diagnosed with low back or lower extremity pain between 2008 and 2015, only 1.2% received surgery, but this subset of patients accounted for 29.3% of total 12-month costs. Many received care inconsistent with guidelines, such as early imaging.

**Meaning:**

The findings suggest that few patients with new episodes of low back or lower extremity pain progress to surgery, and overimaging of low back or lower extremity pain is associated with a significant, avoidable cost burden for a condition that is typically self-limited.

## Teaser

This cohort study examines health care utilization and expenditures among US adult patients with newly diagnosed low back pain or lower extremity pain treated surgically vs nonsurgically.

## Introduction

Up to 80% of the US population will experience low back pain (LBP) or lower extremity pain (LEP) at least once in their lifetime.^1^ These conditions are common reasons for physician visits.^2^ Acute LBP is usually a self-limited condition. Most patients will experience symptomatic resolution irrespective of treatment.^3,4^ Although current guidelines recommend nonpharmacological and conservative measures,^5^ the costs of care associated with LBP and LEP remain significant. In 2013, spinal conditions, defined as both neck and back pain, accounted for the third largest portion of total national health spending after diabetes and ischemic heart disease.^6^

Identifying practice patterns that unnecessarily increase health care spending in patients with LBP and LEP will inform national health care policy. Guidelines of LBP and LEP management discourage use of defined health care services, deeming them potentially unnecessary.^5,7,8,9,10,11,12,13^ Most guidelines agree that imaging should be reserved to identify surgical candidates, recommending that imaging be avoided within 30 days of diagnosis or without exhausting conservative treatment options, such as physical therapy, first.^7,8,10,11,13,14,15^ Patients with LBP and LEP often receive treatments that are neither standardized nor evidence based.^16,17^ Such heterogeneity of treatment patterns makes analysis of health service use challenging.

We present a retrospective cohort study of newly diagnosed, opiate-naive patients with LBP and LEP in the United States with the goal of assessing costs of care and treatment variation in this population. Because management may differ for chronic LBP or LEP, we restricted our analysis to patients with new diagnoses of LBP or LEP; patients with chronic LBP or LEP may have more complex history and, thus, be more likely to receive medical and surgical intervention.^18^ Within this patient population with acute LBP or LEP, we compare detailed cost information between patients who receive surgery and those who do not, using longitudinal follow-up data from the time of their diagnosis. This study characterizes the total health care costs of patients who receive care that is inconsistent with guidelines for the conservative management of LBP and LEP.

## Methods

### Data Source

We used the Truven Health MarketScan Commercial Claims and Encounters and Medicare Supplemental and Coordination of Benefits databases encompassing data from January 1, 2007, to December 31, 2016. The MarketScan Commercial Claims and Encounters database is a collection of commercial inpatient, outpatient, and pharmaceutical claims of more than 75 million employees, retirees, and dependents representing a substantial portion of the US population covered by employer-sponsored insurance. The MarketScan Medicare Supplemental and Coordination of Benefits database contains data from retirees with employer-sponsored medical supplemental insurance. The MarketScan databases, which use a convenience sampling methodology, contain codes from the *International Classification of Diseases, Ninth Revision, Clinical Modification* (*ICD-9-CM*); *International Classification of Diseases, 10th Revision, Clinical Modification *(*ICD-10-CM*); *Current Procedural Terminology*; diagnosis related group; and the National Drug Code.

As this study includes only analysis of secondary deidentified data, it was not considered human subjects research and received exemption from the institutional review board approval at our institution. This study followed the Strengthening the Reporting of Observational Studies in Epidemiology (STROBE) reporting guideline.

### Study Design and Cohort Definition

We designed a retrospective longitudinal cohort study using the MarketScan databases described above. Our study cohort included adult patients (aged ≥18 years) who were newly diagnosed with LBP or LEP between 2008 and 2015 and opiate naive for at least the 6 months before the diagnosis. Opiate-naive patients were defined as those who had not filled a prescription for opiates in the 6 months prior to diagnosis without accounting for days of supply.^19^ As up to 40% of patients with LBP receive opiates within the first year of diagnosis,^19^ this inclusion criterion was created to minimize the risk of misclassifying chronic LBP or LEP as a new diagnosis and exclude patients with chronic pain, for whom different management may be indicated. The initial visit, which served as a proxy for time of LBP or LEP onset, was defined as the first visit at which an enrollee had a claim with an *ICD-9-CM* (or *ICD-10-CM*) code that met the criteria of LBP or LEP (eTable 1 in the Supplement) without any of these inclusion diagnoses in the 12 months prior to this visit. A 12-month continuous enrollment window before and after the initial visit was required for inclusion. We censored all study participants at 12 months. Data analysis was performed between October 6, 2018, and March 7, 2019.

**Figure 1.**
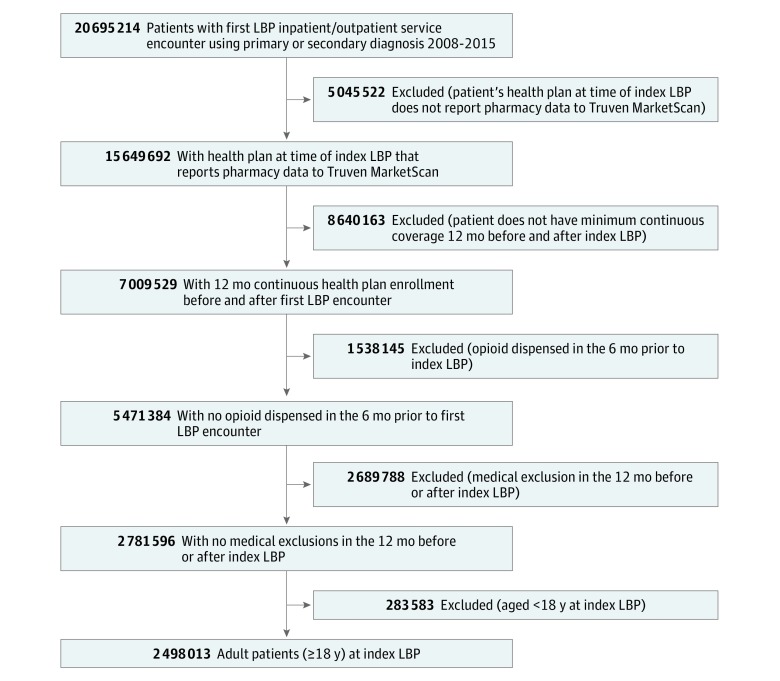
Flow of Cohort Selection LBP indicates low back pain.

Exclusion criteria included presence of a red-flag diagnosis (eg, cauda equina syndrome, foot drop, history of cancer, or infection)^15,20^ at any point in the 12 months before or after the initial visit. Codes from *ICD-9-CM* and *ICD-10-CM* for red-flag diagnoses are included in eTable 1 in the Supplement. Patients with an opiate prescription in the 6 months prior to the initial visit were also excluded. We identified opiate prescriptions (specifically, substances categorized as Drug Enforcement Administration Schedule II or III or Therapeutic Class 60 or 61) from pharmaceutical claims data using variables mapped from the American Hospital Formulary Service Classification Compilation Therapeutic Class.^21^ Figure 1 shows our cohort selection process. The eFigure in the Supplement shows a cohort definition schematic.

### Outcomes and Covariates

The primary outcome measure in this study was a continuous variable denoting total health care spending at 6 and 12 months following a new diagnosis of LBP or LEP. We reported costs according to whether patients received spinal surgery, pattern of conservative management, and types of health care services used. Costs were defined as total eligible charges prior to reductions for deductibles, copayments, and other savings, including both patient and health plan share of payments. Outpatient prescription drug payments were included in the total costs. We use the terms *nonsurgical spending* and *surgical spending* to refer to aggregate costs from the nonsurgical and surgical cohorts, respectively. Based on this definition, costs associated with nonsurgical interventions (eg, imaging) for patients who eventually underwent surgery were included in surgical spending.

We also examined the potential impact of deviation from LBP management guidelines among patients who did not received surgery. For this purpose, we selected the following 2 measurable examples of LBP management that are recommended by multiple published guidelines^7,8,10,11,13,14,15^: (1) imaging should not be obtained within 30 days of diagnosis and (2) imaging should not be obtained without or before a trial of physical therapy (PT).

Patient-level covariates included age in years, sex, comorbidities (based on a validated update to the Elixhauser Comorbidity Index), visits to health professionals, types of spine imaging obtained, other procedures related to LBP or LEP diagnoses (epidural steroid injection [ESI], PT, chiropractic manipulative treatment), spinal surgery (ie, surgery or no surgery), type(s) of surgery received (if applicable), patient’s geographic location, and insurance plan type. Spinal surgery was defined as a static, rather than time-varying, variable (eg, if patients received surgery at 6 months after diagnosis, they were considered patients treated surgically throughout their follow-up period rather than classified as patients treated nonsurgically before surgery and recategorized as patients treated surgically afterward).

### Statistical Analysis

We used χ^2^ tests to compare differences between surgical and nonsurgical cohorts and between guideline-adherent and nonadherent groups for categorical variables (health professional visits, imaging studies, procedures). Differences between continuous variables were assessed with paired *t* tests (unadjusted costs) or Wilcoxon rank sum tests (age). For cost comparison between nonsurgical and surgical cohorts, and between guideline-adherent and nonadherent groups within the nonsurgical cohort, we ran multivariate regression models adjusting for age groups, sex, and comorbidities. Regression-adjusted means were estimated using the margins command in Stata. Additionally, nonsurgical and surgical cohorts were matched for age groups, sex, and comorbidities with coarsened exact matching, a method that is similar to propensity score matching but reduces model dependence and estimation error.^22,23^ All tests were 2-sided and conducted at the .05 level of significance. All analyses were performed with SAS statistical software version 9.4 (SAS Institute Inc) and Stata statistical software version 14 (StataCorp LLC).

## Results

### Cohort Demographic Characteristics

We identified 2 498 013 opiate-naive patients (median [interquartile range] age, 47 [36-58] years; 1 373 076 [55.0%] female) with a new diagnosis of LBP or LEP without a red-flag diagnosis between 2008 and 2015. We divided these patients based on the receipt of surgical intervention. The vast majority of patients (98.8%) did not receive surgery within the 12 months following diagnosis. Compared with the nonsurgical cohort, patients who received surgery were slightly older (median [interquartile range] age, 51 [41-60] years vs 47 [36-58] years; difference, −3.3 years; 95% CI, −3.5 to −3.1 years; *P* < .001) and less likely to be female (41.5% vs 55.1%; difference, −13.6%; 95% CI, −14.2% to −13.1%; *P* < .001). Both groups consisted of young, privately insured patients who were healthy prior to their episode of LBP or LEP, with 78.5% of patients having no or 1 preexisting comorbidity. Detailed patient demographic information across both groups is presented in Table 1 and eTable 2 in the Supplement.

**Table 1.** Descriptive Characteristics and Health Care Utilization Among 2 498 013 Patients With Newly Diagnosed Low Back or Lower Extremity Pain, 2008 to 2015

Characteristic	No Surgery (n = 2 467 389)	Surgery (n = 30 624)	*P* Value
Age group, No. (%), y			
18-29	354 322 (14.4)	1920 (6.3)	NA
30-39	444 613 (18.0)	4800 (15.7)	
40-49	566 910 (23.0)	7597 (24.8)	
50-59	586 459 (23.8)	8530 (27.9)	
60-69	312 076 (12.7)	5198 (17.0)	
70-79	122 976 (5.0)	2092 (6.8)	
≥80	80 033 (3.2)	487 (1.6)	
Age, median (interquartile range) y	47 (36-58)	51 (41-60)	<.001
Female, No. (%)	1 360 360 (55.1)	12 716 (41.5)	<.001
No. of Elixhauser comorbidities, No. (%)			
0	1 317 451 (53.4)	15 688 (51.2)	<.001
1	620 272 (25.1)	7949 (26.0)	
≥2	529 666 (21.6)	6987 (22.8)	
Common Elixhauser comorbidities, No. (%)			
Hypertension, uncomplicated	580 363 (23.5)	8557 (27.9)	NA
Diabetes, uncomplicated	227 808 (9.2)	3450 (11.3)	
Depression	192 190 (7.8)	2270 (7.4)	
Health professional visits at any point in time after diagnosis, No. (%)			
Primary care practitioner	1 719 243 (69.7)	28 474 (93.0)	<.001
Chiropractor	308 941 (12.5)	2849 (9.3)	<.001
Physical therapist	170 764 (6.9)	7955 (26.0)	<.001
Imaging at any point in time post diagnosis, No. (%)			
Lumbar computed tomography	24 604 (1.0)	3751 (12.3)	<.001
Lumbar magnetic resonance imaging	295 632 (12.0)	26 304 (85.9)	<.001
Lumbar radiograph	740 845 (30.0)	22 327 (72.9)	<.001
Procedures at any point in time post diagnosis, No. (%)			
Epidural steroid injection	86 994 (3.5)	12 274 (40.1)	<.001
Physical therapy	335 792 (13.6)	12 646 (41.3)	<.001
Chiropractic manipulative treatment	301 862 (12.2)	2660 (8.7)	<.001
Types of surgery received, No. (%)			
Anterior	NA	1924 (6.3)	NA
Instrumentation	NA	8220 (26.8)	
Multilevel	NA	8244 (26.9)	
Posterior	NA	29 421 (96.1)	
6-mo cost, $			
Unadjusted per patient, mean (SD)	655 (1716)	17 640 (24 562)	<.001
Regression-adjusted, unmatched, mean (SD)	656 (2.0)	17 624 (18.4)	<.001
Regression-adjusted, matched, mean (SD)	676 (2.1)	17 640 (18.6)	<.001
Collective sum of group (% of total cost)	1 616 604 470 (75.0)	540 098 155 (25.0)	NA
12-mo cost, $			
Unadjusted per patient, mean (SD)	769 (2093)	25 613 (29 917)	<.001
Regression-adjusted, unmatched, mean (SD)	769 (2.5)	25 596 (22.4)	<.001
Regression-adjusted, matched, mean (SD)	795 (2.5)	25 613 (22.6)	<.001
Collective sum of group (% of total cost)	1 896 529 067 (70.8)	784 103 292 (29.3)	NA

Abbreviation: NA, not applicable.

### Health Care Utilization

More than half (55.7%) of all study patients received no intervention. Patients in the nonsurgical cohort were less likely to visit primary care practitioners (69.7% vs 93.0%; difference, −23.3%; 95% CI, −23.6% to −23.0%; *P* < .001) and physical therapists (6.9% vs 26.0%; difference, −19.1%; 95% CI, −19.5% to −18.6%; *P* < .001) but more likely to visit chiropractors (12.5% vs 9.3%; difference, 3.2%; 95% CI, 2.9%-3.5%; *P* < .001). Compared with the nonsurgical group, patients in the surgical group received more radiological imaging throughout the management of their condition, including lumbar computed tomography (12.3% vs 1.0%; difference, −11.3%; 95% CI, −11.6% to −10.9%; *P* < .001), magnetic resonance imaging (85.9% vs 12.0%; difference, −73.9%; 95% CI, −74.3% to −73.5%; *P* < .001), and radiography (72.9% vs 30.0%; difference, −42.9%; 95% CI, −43.4% to −42.4%; *P* < .001). This group also had more frequent use of ESI (40.1% vs 3.5%; difference, −36.6%; 95% CI, −37.1% to −36.0%; *P* < .001) and PT (41.3% vs 13.6%; difference, −27.7%; 95% CI, −28.2% to −27.1%; *P* < .001) during the follow-up period after the diagnosis (Table 1).

Collectively, expenditures attributable to patients treated nonsurgically, who formed the majority (98.8%) of our study population, accounted for 75% of the total 6-month spending for LBP and LEP care ($1.6 billion). The remaining 25% of the total 6-month cost ($540 million) was spent among the 1.2% patients who underwent surgery. After matching for age, sex, and comorbidity, per-patient 6-month cost was significantly higher for the surgical cohort compared with the nonsurgical cohort ($17 640 [95% CI, $17 604-$17 676] vs $676 [95% CI, $672-$680]; *P* < .001).

A similar pattern was observed with costs over the 12 months after diagnosis. The nonsurgical cohort accounted for 70.8% of the total spending ($1.8 billion). The remaining 29.3% of the total 12-month cost was spent among the surgical cohort ($784 million). Age-, sex-, and comorbidity-matched per-patient 12-month cost was significantly higher for the surgical cohort ($25 613 [95% CI, $25 569-$25 657] vs $795 [95% CI, $790-$800]; *P* < .001) (Table 1).

### Guideline Adherence in the Nonsurgical Cohort

Approximately one-third of patients (32.3%) treated nonsurgically received imaging within 30 days of diagnosis (computed tomography, 0.7%; magnetic resonance imaging, 8.1%; and radiography, 26.7%). Adjusted mean 12-month costs among patients who received imaging within 30 days of diagnosis were more than 2 times greater than costs for patients who did not receive early imaging ($1194 [95% CI, $1190-$1199] vs $566 [95% CI, $563-$569]; *P* < .001). This association was true for all imaging modalities, including computed tomography ($2244 [95% CI, $2212-$2275] vs $758 [95% CI, $756-$761]; *P* < .001), magnetic resonance imaging ($2399 [95% CI, $2390-$2408] vs $625 [95% CI, $622-$628]; *P* < .001), and radiography ($992 [95% CI, $987-$998] vs $687 [95% CI, $684-$690]; *P* < .001) (Table 2).

**Table 2.** Mean 12-Month Costs Based on Guideline Adherence Among 2 467 389 Patients Who Did Not Receive Surgery

Service Use Discouraged by Guidelines	Patients, No. (%)		Unadjusted Mean 12-mo Cost per Patient, $			Regression-Adjusted Mean 12-mo Cost per Patient, $		
	Adherent^a^	Nonadherent^a^	Adherent^a^	Nonadherent^a^	*P* Value	Adherent^a^	Nonadherent^a^	*P* Value
Imaging within 30 d of diagnosis								
Any imaging	1 671 419 (67.7)	795 970 (32.3)	561 (1803)	1204 (2546)	<.001	566 (1.6)	1194 (2.3)	<.001
Lumbar computed tomography	2 450 225 (99.3)	17 164 (0.7)	758 (2064)	2300 (4412)	<.001	758 (1.3)	2244 (16.0)	<.001
Lumbar magnetic resonance imaging	2 267 341 (91.9)	200 048 (8.1)	624 (1835)	2413 (3597)	<.001	625 (1.4)	2399 (4.6)	<.001
Lumbar radiograph	1 808 694 (73.3)	658 695 (26.7)	683 (2027)	1004 (2249)	<.001	687 (1.6)	992 (2.6)	<.001
Imaging without or before physical therapy								
Any imaging	1 597 129 (64.7)	870 260 (35.3)	505 (1623)	1253 (2689)	<.001	509 (1.6)	1245 (2.2)	<.001
Lumbar computed tomography	2 444 400 (99.1)	22 989 (0.9)	750 (2006)	2803 (6199)	<.001	750 (1.3)	2751 (13.8)	<.001
Lumbar magnetic resonance imaging	2 201 890 (89.2)	265 499 (10.8)	563 (1693)	2472 (3700)	<.001	565 (1.4)	2463 (3.9)	<.001
Lumbar radiograph	1 751 666 (71.0)	715 723 (29.0)	649 (1884)	1063 (2509)	<.001	653 (1.6)	1052 (2.5)	<.001
Imaging within 30 d of diagnosis or imaging without or before physical therapy	Neither: 1 611 988 (64.5)	Either: 886 025 (35.5)	Neither: 964 (5587)	Either: 1272 (2713)	<.001	980 (3.8)	1244 (5.1)	<.001

^a^ Patients whose treatment was adherent did not receive the services listed; those whose treatment was nonadherent did receive the services.

We then evaluated the costs associated with another example of guideline nonadherence, imaging without or before PT. Compared with the 35.3% of patients treated nonsurgically who received imaging without or prior to PT, patients who did undergo PT prior to receiving any imaging accrued lower adjusted mean 12-month costs ($1245 [95% CI, $1240-$1249] vs $509 [95% CI, $506-$513]; *P* < .001). Similarly, patients whose treatment was compliant with both recommendations (ie, no imaging within 30 days and no imaging without PT) constituted 64.5% of the nonsurgical cohort and had lower 12-month mean spending than the rest of the nonsurgical cohort ($980 [95% CI, $973-$988] vs $1242 [95% CI, $1232-$1252]; *P* < .001) (Table 2). Rates of guideline deviation for each year of diagnosis showed a downward trend from 2008 (37%) to 2015 (33.1%) (eTable 3 in the Supplement). Analysis of additional descriptive characteristics between guideline-adherent and nonadherent groups is available in eTable 4 in the Supplement. Patients who underwent imaging tests early or before PT tended to be older (mean age 50.1 vs 45.9 years; difference 4.16 years; 95% CI, 4.12-4.20 years; *P* < .001) and have more comorbidities (≥2 comorbidities: 24.8% vs 19.7%).

### Cost Breakdown of the Nonsurgical Cohort

We characterized the distribution of interventions and the associated 12-month costs in patients with LBP or LEP who underwent surgical vs nonsurgical treatment. For each category of intervention, we calculated the aggregate value of their 12-month costs as a group and also the percentage of its dollar amount in the context of the total nonsurgical or surgical spending and the total spending (nonsurgical and surgical spending combined).

**Figure 2.**
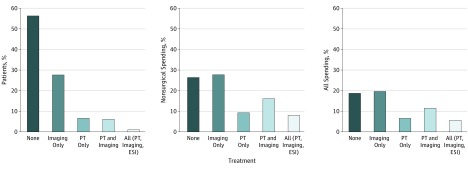
Number of Patients and Sum of Total 12-Month Costs Based on Different Management Patterns for 2 467 389 Patients in the Nonsurgical Cohort ESI indicates epidural steroidal injection; PT, physical therapy.

Among patients treated nonsurgically, 56.3% did not receive PT, imaging, or ESI. They accounted for 26.3% of the total 12-month health care expenditures ($498 million). Patients who received imaging only (27.6%) accounted for 27.7% and 19.6% of the total 12-month cost in the nonsurgical and entire cohorts, respectively ($525 million). Physical therapy alone without imaging or ESI (6.5% of total PT) accounted for 9.2% of the total 12-month spending ($174 million). The mean 12-month spending per patient increased with greater use of services (PT, imaging, and ESI: $5868; PT and imaging: $2056; PT only: $1090; imaging only: $770; none: $359) (Figure 2).

### Cost Breakdown of the Surgical Cohort

**Figure 3.**
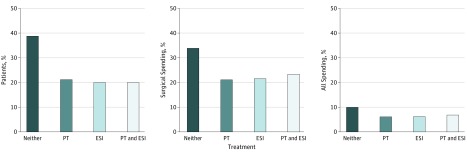
Number of Patients and Sum of Total 12-Month Costs Based on Different Management Patterns for 30 624 Patients in the Surgical Cohort ESI indicates epidural steroidal injection PT, physical therapy.

The same analysis was performed for the surgical cohort, but only PT and ESI services were included. This assumed surgery entailed some form of preoperative imaging. Among patients treated surgically, 38.7% received neither PT nor ESI. These patients had health care expenditures of $265 million as a group (mean per patient, $22 386) during the first 12 months after diagnosis. Aggregate 12-month costs from patients who underwent surgery and received PT only (21.2%) amounted to $166 million, with per-patient costs of $25 590. Patients who underwent surgery and received ESI without PT (20.0%) collectively had a total 12-month cost of $169 million (mean per patient, $27 578) and those who had both PT and ESI (20.1%) spent $183 million as a group (mean per patient, $299,00) during the first 12-month period (Figure 3).

## Discussion

Low back pain with or without LEP is highly prevalent and associated with a significant cost burden.^2,24^ This is the first study, to our knowledge, to follow the trajectories of a large sample of patients with LBP and LEP across the United States to characterize the costs associated with LBP and LEP management. Based on a nationwide sample of nearly 2.5 million patients from a large 7-year database, this assessment presents generalizable patterns in LBP care that transcend variations in individual practices. We additionally identify certain management patterns of LBP or LEP that may serve as potential targets for reducing health care expenditures. Unlike previous studies^25,26,27,28^ on costs of LBP that have focused on surgical management, this study also suggests that nonsurgical management of LBP may be further improved with guideline adherence.

Our data show that deviation from management guidelines is common and costly. Among patients treated nonsurgically, those who received early imaging, defined as imaging within 30 days of diagnosis (32.3% of patients treated nonsurgically), or obtained imaging without physical therapy (35.3%) accrued higher costs of care compared with patients whose condition was managed according to guideline recommendations. Imaging alone without further interventions (27.6%) was also expensive, accounting for nearly 20% of total nonsurgical health care expenditures, a total of more than half a billion dollars annually. In addition to guideline nonadherence, surgery was also associated with spending for LBP or LEP care. A small proportion (1.2%) of opiate-naive patients with newly diagnosed LBP or LEP underwent surgical management, but this subset of patients accounted for nearly 30% of the total expenditures tracked in this assessment.

### Nonsurgical Management and Guideline Nonadherence

Our study population comprised opiate-naive patients newly presenting with LBP or LEP without red-flag diagnoses. These patients were generally young and healthy with a low comorbidity burden, a patient population that should not consume significant health care resources. Most patients in our study (55.7%) received no interventions—no PT, ESIs, imaging, or surgery—in keeping with generally benign and self-limited nature of episodes of LBP and LEP.^3,4^

When nonoperative care was offered, however, it frequently did not follow established guidelines. Multiple guidelines exist for LBP management,^5,7,8,9,10,11,12,13^ and most recommend against overuse of imaging in patients who are not candidates for surgery and caution against early use of imaging before a trial of other nonsurgical treatment.^10,11,13,14,15^ Despite these general practice guidelines, almost one-third of patients treated nonsurgically (32.3%) received imaging within 30 days of LBP or LEP diagnosis, and a similar portion of patients (35.3%) underwent imaging without trying PT first.

Such deviations from guidelines were associated with significantly greater spending: adjusted mean 12-month costs were 2-fold higher among patients who underwent imaging in a manner inconsistent with guidelines compared with guideline-adherent treatment (imaging within 30 days: $1194 [95% CI, $1190-$1199] vs $566 [95% CI, $563-$569]; *P* < .001; imaging without or before PT: $509 [95% CI, $506-$513] vs $1245 [95% CI, $1240-$1249]; *P* < .001). The cost of imaging itself is the most intuitive explanation for the increased expenditure, although imaging findings may have also indirectly raised the cost by increasing the likelihood of receiving other nonsurgical treatments such as ESI. The high cost associated with lumbar imaging was previously investigated in a study^29^ of Medicare patients with newly diagnosed LBP. The authors estimated annual savings of $362 million if these patients were managed conservatively according to clinical guidelines and did not obtain imaging during the first 6 weeks of diagnosis.

Patients who underwent imaging early or before PT tended to be older and have more comorbidities, but many patients who underwent early imaging had no further interventions. Patients treated nonsurgically who received imaging without PT, ESI, or surgery (27.6%) had an aggregate 12-month cost of $525 million, accounting for a significant, possibly avoidable, portion of the total 12-month nonsurgical spending in our patient cohort (19.6%). Considering that these patients underwent no further interventions, it is possible this imaging did not change management of this self-limited condition.

### Surgical Management

In our study cohort, only a small proportion (1.2%) of patients with newly diagnosed LBP or LEP had surgical intervention in the first 12 months after presentation. Although the vast majority of our study population did not undergo surgical intervention, the few patients who underwent surgery accounted for a substantial portion of costs associated with LBP and LEP care. This may reflect more severe symptoms in patients eventually requiring operative treatment. Surgically treated patients also underwent various other interventions including PT and ESIs. These patients likely have episodes of LBP or LEP with longer durations that do not spontaneously resolve.

Inability to reliably identify patients who will respond to surgical treatment has resulted in large variability in practice.^27^ Literature reviews have reported 6- to 10-fold variations in rates of spinal surgery among US cities depending on practice duration, operative volume, and practice model.^25,26^

Lack of standardization in the management of the conditions of patients who underwent surgery was also evident in our study. Among patients who had surgery, 21.2% received PT and 20.1% received both ESI and PT, whereas 38.7% received neither PT nor ESI. Although surgery was likely indicated for some patients, our results suggest that many patients receive surgery without undergoing a trial of conservative treatment, contrary to the recommendations of multiple studies.^3,10,13,29,30^ Increased use of imaging, which has been associated with higher rates of spinal surgery in previous studies,^29,31^ may partly explain surgical intervention offered early in some patients’ disease course.

### Limitations

As with all large database studies, interpretations of this study are limited by the administrative nature of the data. We assumed accuracy of all diagnosis and procedure codes in the database. Because our database lacks granular clinical information, our findings speak to the appropriateness of management patterns at a population level rather than an individual level. However, the generalizability of the large privately insured patient population is one of our study strengths. The MarketScan database compiles mostly private insurance claims; hence, our data do not reflect all treatments sought out by patients with LBP or LEP, particularly several nonmedical, alternative therapy options (eg, acupuncture, massage, and yoga) that may have some efficacy.^8,9,10,11^ Symptom onset was defined as when the patient first manifested LBP and LEP diagnostic codes after an encounter with a health care professional. This estimate is susceptible to bias, as patients’ symptoms may have begun at variable times prior to the initial visit, and we may have overestimated the rate of guideline nonadherence. However, in our cohort selection, we have mandated a minimum of 12-month continuous enrollment prior to diagnosis and excluded patients with opiate use in the 6 months preceding the index visit. Although this approach does not entirely mitigate the risk of misclassification of acute LBP given the prevalence of the condition, our careful cohort selection eliminated any patients with pain severe enough to require opiates or prompt a physician visit for at least 1 year in addition to those with recent insurance change. Because we lacked access to a severity index of the disease (eg, standardized pain scores), we were unable to determine the extent to which the increased costs associated with surgery or early imaging can be explained by the severity of presenting symptoms (ie, patients treated nonsurgically may have had less severe LBP and required fewer health care resources).

## Conclusions

Guideline nonadherence and spinal surgery in patients with newly diagnosed LBP or LEP are associated with a significant economic burden in the United States. Patients who obtain early imaging or receive surgery for LBP and LEP without exhausting conservative therapies account for a disproportionate amount of total costs associated with this common condition.

## Notes

Supplement. **eFigure.** A Schematic Illustrating Study Cohort Definition **eTable 1.** ICD-9-CM and ICD-10 CM Codes for Inclusion and Exclusion **eTable 2.** Additional Descriptive Baseline Characteristics of Patients With Newly Diagnosed Low Back or Lower Extremity Pain, 2008-2015 (N = 2,498,013) **eTable 3.** Guideline Adherence Among Non-surgical Patients by Year of Diagnosis (N = 2,401,204) **eTable 4.** Descriptive Characteristics and Health Care Service Usage Based on Guideline Adherence Among Non-surgical Patients (N = 2,401,204)
