# Supplementary material for: Expenditures and Health Care Utilization Among Adults With Newly Diagnosed Low Back and Lower Extremity Pain
Source: JAMA Netw Open. 2019 May 10;2(5):e193676. doi: 10.1001/jamanetworkopen.2019.3676 (PMC6512284; doi:10.1001/jamanetworkopen.2019.3676)
Supplement: Supplement. — eFigure. A Schematic Illustrating Study Cohort Definition eTable 1. ICD-9-CM and ICD-10 CM Codes for Inclusion and Exclusion eTable 2. Additional Descriptive Baseline Characteristics of Patients With Newly Diagnosed Low Back or Lower Extremity Pain, 2008-2015 (N = 2,498,013) eTable 3. Guideline Adherence Among Non-surgical Patients by Year of Diagnosis (N = 2,401,204) eTable 4. Descriptive Characteristics and Health Care Service Usage Based on Guideline Adherence Among Non-surgical Patients (N = 2,401,204) [file jamanetwopen-2-e193676-s001.pdf]

Supplementary Online Content

Kim LH, Vail D, Azad TD, et al. Expenditures and health care utilization among adults with newly diagnosed low back and lower extremity pain. *JAMA Netw Open*. 2019;2(5):e193676.
doi:10.1001/jamanetworkopen.2019.3676

eFigure. A Schematic Illustrating Study Cohort Definition

eTable 1. ICD-9-CM and ICD-10 CM Codes for Inclusion and Exclusion

eTable 2. Additional Descriptive Baseline Characteristics of Patients With Newly Diagnosed Low Back or Lower Extremity Pain, 2008-2015 (N = 2,498,013)

eTable 3. Guideline Adherence Among Non-surgical Patients by Year of Diagnosis (N = 2,401,204)

eTable 4. Descriptive Characteristics and Health Care Service Usage Based on Guideline Adherence Among Non-surgical Patients (N = 2,401,204)

This supplementary material has been provided by the authors to give readers additional information about their work.

© 2019 Kim LH et al. *JAMA Network Open.*

eFigure. A schematic illustrating study cohort definition

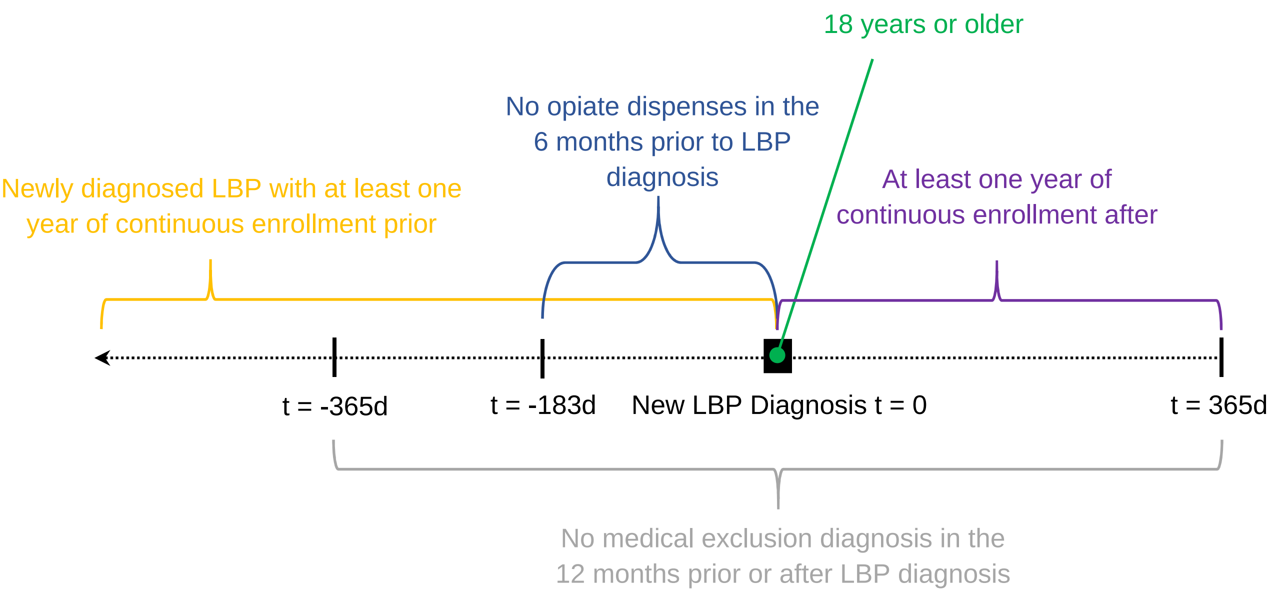

LBP, low back pain.

© 2019 Kim LH et al. *JAMA Network Open.*

eTable 1. ICD-9-CM and ICD-10 CM codes for inclusion and exclusion

	Codes	Description
Inclusion	721.3, M47.817	Lumbosacral spondylosis without myelopathy
	721.4, M47.14, M47.16	Thoracic or lumbar spondylosis with myelopathy
	722.1, M51.26, M51.27	Displacement of lumbar or lumbosacral intervertebral disc
	722.52, M51.36, M51.37	Degeneration of lumbar or lumbosacral intervertebral disc
	724.2	Lumbago
	724.3, M54.30	Sciatica
	724.4	Thoracic or lumbosacral neuritis or radiculitis, unspecified
	724.5	Backache, unspecified
	738.4, M43.00	Spondylolysis, site unspecified
	M43.10	Spondylolisthesis, site unspecified
	M54.14-M54.17	Radiculopathy (thoracic, thoracolumbar, lumbar, or lumbosacral)
	M54.89, M54.9	Dosalgia
Red flag diagnoses	344.6-344.69, G83.4	Cauda equina syndrome
	736.79	Foot drop
	720.0-720.9, M45	Ankylosing spondylitis
	038-038.99, 995.9-995.99, A40, A41, R65	Septicemia; SIRS (Sepsis)
	788.3-788.39, N39.3, N39.4, R32	Urinary incontinence
	787.6-787.69, R15	Bowel incontinence
	805.4, 805.5, 806.4, 806.5, 839.20, 739.3, 839.30, 847.2, S32, S33.0, S33.1, S33.5	Lumbar sprains, fractures, and dislocations
	334.1, 342-342.99, 343-343.99, 344–344.6, 344.9, G80, G81, G11.4	Paralysis
	338.2, G89.2	Chronic pain
	140-239, C00-D49	Cancer

© 2019 Kim LH et al. *JAMA Network Open.*

eTable 2. Additional descriptive baseline characteristics of patients with newly diagnosed low back or lower extremity pain, 2008-2015 (N = 2,498,013)

	**No Surgery (N = 2,467,389)**	**Surgery (N = 30,624)**
**Region**		
Northeast	369,475 (16.8%)	3,755 (13.9%)
North Central	506,980 (23.1%)	7,265 (26.9%)
South	882,901 (40.2%)	11,855 (44.0%)
West	407,828 (18.6%)	3,765 (14.0%)
Unknown	28,417 (1.3%)	328 (1.2%)
**Health plan type**		
Preferred provider organization (PPO)	1,304,146 (61.6%)	16,714 (64.7%)
Health maintenance organization (HMO)	327,779 (15.5%)	2,805 (11.0%)
Point of service (POS)	160,914 (7.6%)	1,996 (7.7%)
Consumer directed health plans (CDHP)	152,691 (7.2%)	1,894 (7.3%)
High deductible health plan (HDHP)	79,043 (3.7%)	1,044 (4.0%)
Comprehensive	51,823 (2.5%)	890 (3.4%)
Exclusive provider organization (EPO)	51,823 (1.3%)	304 (1.2%)
POS with capitation	13,413 (0.6%)	165 (0.6%)
**Year**		
2008	336,228 (13.6%)	4,822 (15.8%)
2009	356,585 (14.5%)	4,845 (15.8%)
2010	351,387 (14.2%)	4,615 (15.1%)
2011	370,908 (15.0%)	4,662 (15.2%)
2012	321,587 (13.0%)	3,817 (12.5%)
2013	279,361 (11.3%)	3,262 (10.7%)
2014	225,395 (9.1%)	2,556 (8.4%)
2015	225,938 (9.2%)	2,045 (6.7%)

© 2019 Kim LH et al. *JAMA Network Open.*

eTable 3. Guideline adherence among non-surgical patients by year of diagnosis (N = 2,401,204)

Year of diagnosis	(1) Imaging within 30 days of diagnosis		(2) Imaging without/before physical therapy		(1) or (2)	
	Adherent/No	Non-Adherent/Yes	Adherent/No	Non-Adherent/Yes	Adherent/Neither	Non-Adherent/Either
2008	223,432 (66.5%)	112,796 (33.6%)	211,815 (63%)	124,413 (37.0%)	214,867 (63%)	126,183 (37%)
2009	236,924 (66.4%)	119,661 (33.6%)	225,733 (63.3%)	130,852 (36.7%)	228,418 (63.2%)	133,012 (36.8%)
2010	235,303 (67.0%)	116,084 (33.0%)	225,011 (64.0%)	126,376 (36.0%)	227,268 (63.8%)	128,734 (36.2%)
2011	249,469 (67.3%)	121,439 (32.7%)	238,516 (64.3%)	132,392 (35.7%)	240,756 (64.1%)	134,814 (35.9%)
2012	219,350 (68.2%)	102,237 (31.8%)	210,032 (65.3%)	111,555 (34.7%)	211,742 (65.1%)	113,662 (34.9%)
2013	191,507 (68.6%)	87,854 (31.5%)	183,662 (65.7%)	95,699 (34.3%)	184,977 (65.5%)	97,646 (34.6%)
2014	157,051 (69.7%)	68,344 (30.3%)	150,367 (66.7%)	75,028 (33.3%)	151,514 (66.5%)	76,437 (33.5%)
2015	158,383 (70.0%)	67,555 (30.0%)	151,993 (67.3%)	73,945 (32.7%)	152,446 (66.9%)	75,537 (33.1%)

Values are expressed as n (%).

© 2019 Kim LH et al. *JAMA Network Open.*

eTable 4. Descriptive characteristics and health care service usage based on guideline adherence among non-surgical patients (N = 2,401,204)

	(1) Imaging within 30 days of diagnosis			(2) Imaging without/before physical therapy			(1) or (2)		
	Adherent/No	Non-Adherent/Yes	*P*	Adherent/No	Non-Adherent/Yes	*P*	Adherent/ Neither	Non-Adherent/ Either	*P*
Age group (years)			-			-			-
18-29	263,557 (15.8%)	90,765 (11.4%)		256,517 (16.1%)	97,805 (11.2%)		256,229 (15.9%)	100,013 (11.3%)	
30-39	324,442 (19.4%)	120,171 (15.1%)		313,460 (19.6%)	131,153 (15.1%)		314,821 (19.5%)	134,592 (15.2%)	
40-49	394,531 (23.6%)	172,379 (21.7%)		378,181 (23.7%)	188,729 (21.7%)		381,732 (23.7%)	192,775 (21.8%)	
50-59	386,921 (23.2%)	199,538 (25.1%)		367,092 (23.0%)	219,367 (25.2%)		371,935 (23.1%)	223,054 (25.2%)	
60-69	191,335 (11.5%)	120,741 (15.2%)		179,892 (11.35)	132,184 (15.2%)		183,343 (11.4%)	133,931 (15.1%)	
70-79	67,392 (4.0%)	55,584 (7.0%)		61,972 (3.9%)	61,004 (7.0%)		63,614 (4.0%)	61,454 (6.9%)	
80+	43,241 (2.6%)	36,792 (4.6%)		40,015 (2.5%)	40,018 (4.6%)		40,314 (2.5%)	40,206 (4.5%)	
Median age (years)	46 (35-57)	51 (39-61)	<.001	46 (35-56)	51 (39-61)	<.001	46 (35-56)	51 (39-60)	<.001
Sex (female)	936,786 (56.1%)	423,574 (53.2%)	<.001	894,113 (56.0%)	466,247 (53.6%)	<.001	899,324 (55.8%)	473,752 (53.5%)	<.001
Number of Elixhauser comorbidities									
0	921,895 (55.2%)	395,556 (49.7%)		888,724 (55.7%)	428,727 (49.3%)		895,110 (55.5%)	438,029 (49.4%)	
1	416,057 (24.9%)	204,215 (25.7%)		395,650 (24.8%)	224,622 (25.8%)		399,885 (24.8%)	228,336 (25.8%)	
2+	333,467 (19.9%)	196,199 (24.6%)		312,755 (19.5%)	216,911 (24.9%)		316,993 (19.7%)	219,660 (24.8%)	

© 2019 Kim LH et al. *JAMA Network Open.*

Common Elixhauser comorbidities									
Hypertension, uncomplicated	361,274 (21.6%)	219,089 (27.5%)		339,135 (21.2%)	241,228 (27.7%)		344,547 (21.4%)	244,373 (27.6%)	
Diabetes, uncomplicated	139,677 (8.4%)	88,131 (11.1%)		130,904 (8.2%)	96,904 (11.1%)		133,260 (8.3%)	97,998 (11.1%)	
Depression	134,827 (8.1%)	57,363 (7.2%)		127,785 (8.0%)	64,405 (7.4%)		128,906 (8.0%)	65,554 (7.4%)	
Health professional visits at any point in time post diagnosis									
Primary care practitioner	1,189,592 (71.2%)	529,651 (66.5%)	<.001	1,121,795 (70.2%)	597,448 (68.7%)	<.001	1,136,011 (70.4%)	611,706 (69.0%)	<.001
Chiropractor	217,021 (13.0%)	91.920 (11.6%)	<.001	214,659 (13.4%)	94,282 (10.8%)	<.001	208,568 (12.95)	103,222 (11.7%)	<.001
Physical therapist	99,168 (5.9%)	71,596 (9.0%)	<.001	93,881 (5.9%)	76,883 (8.8%)	<.001	97,532 (6.1%)	81,187 (9.2%)	<.001
Procedures at any point in time post diagnosis									
Epidural steroid injection	28,163 (1.7%)	58,831 (7.4%)	<.001	17,883 (1.1%)	69,111 (7.9%)	<.001	28,602 (1.8%)	70,666 (8.0%)	<.001
Physical Therapy	194,786 (11.7%)	141,006 (17.7%)	<.001	196,003 (12.3%)	139,789 (16.1%)	<.001	192,884 (12.0%)	155,554 (17.6%)	<.001
Chiropractic manipulative treatment	213,397 (12.8%)	88,465 (11.1%)	<.001	211,107 (13.2%)	90,755 (10.4%)	<.001	204,958 (12.7%)	99,564 (11.2%)	<.001

Values are expressed as n (%) or median (IQR).

© 2019 Kim LH et al. *JAMA Network Open.*
